# Supplementary material for: Physicochemical and Functional Changes in Lotus Root Polysaccharide Associated with Noncovalent Binding of Polyphenols
Source: Foods. 2023 Mar 1;12(5):1049. doi: 10.3390/foods12051049 (PMC10001286; doi:10.3390/foods12051049)
Supplement: Supplementary file 1 [file foods-12-01049-s001.zip › foods-2198484-supplementary.pdf]

Supplementary information

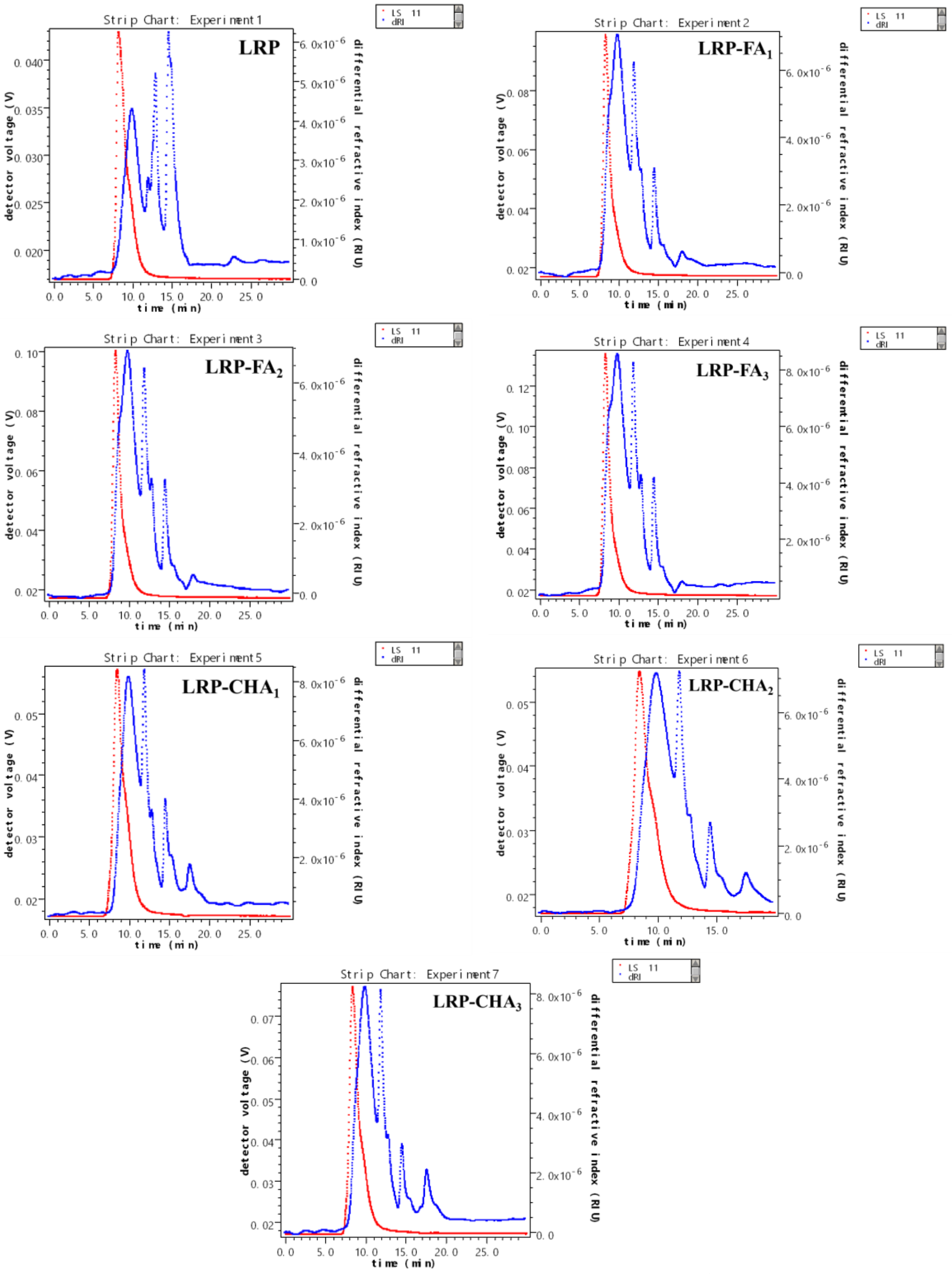

Figure S1. HPSEC-MALLS-RI chromatogram of LRP, and polyphenol complex
